# Contrasting Dependencies of Photosynthetic Capacity on Leaf Nitrogen in Early- and Late-Successional Tropical Montane Tree Species

**DOI:** 10.3389/fpls.2020.500479

**Published:** 2020-09-17

**Authors:** Camille Ziegler, Mirindi Eric Dusenge, Brigitte Nyirambangutse, Etienne Zibera, Göran Wallin, Johan Uddling

**Affiliations:** ^1^ Department of Biological and Environmental Sciences, University of Gothenburg, Gothenburg, Sweden; ^2^ UMR EcoFoG, AgroParisTech, CNRS, CIRAD, INRAE, Université des Antilles, Université de Guyane, Kourou, France; ^3^ Université de Lorraine, AgroParisTech, INRAE, UMR Silva, Nancy, France; ^4^ Department of Biology, University of Rwanda, Huye, Rwanda; ^5^ Department of Biology, The University of Western Ontario, London, ON, Canada

**Keywords:** photosynthesis, nitrogen, allocation, early successional, late successional, tropical montane forests

## Abstract

Differences in photosynthetic capacity among tree species and tree functional types are currently assumed to be largely driven by variation in leaf nutrient content, particularly nitrogen (N). However, recent studies indicate that leaf N content is often a poor predictor of variation in photosynthetic capacity in tropical trees. In this study, we explored the relative importance of area-based total leaf N content (N_tot_) and within-leaf N allocation to photosynthetic capacity versus light-harvesting in controlling the variation in photosynthetic capacity (i.e. *V*_cmax_, *J*_max_) among mature trees of 12 species belonging to either early (ES) or late successional (LS) groups growing in a tropical montane rainforest in Rwanda, Central Africa. Photosynthetic capacity at a common leaf temperature of 25˚C (i.e. maximum rates of Rubisco carboxylation, *V*_cmax25_ and of electron transport, *J*_max25_) was higher in ES than in LS species (+ 58% and 68% for *V*_cmax25_ and *J*_max25_, respectively). While N_tot_ did not significantly differ between successional groups, the photosynthetic dependency on N_tot_ was markedly different. In ES species, *V*_cmax25_ was strongly and positively related to N_tot_ but this was not the case in LS species. However, there was no significant trade-off between relative leaf N investments in compounds maximizing photosynthetic capacity versus compounds maximizing light harvesting. Both leaf dark respiration at 25˚C (+ 33%) and, more surprisingly, apparent photosynthetic quantum yield (+ 35%) was higher in ES than in LS species. Moreover, R_d25_ was positively related to N_tot_ for both ES and LS species. Our results imply that efforts to quantify carbon fluxes of tropical montane rainforests would be improved if they considered contrasting within-leaf N allocation and photosynthetic N_tot_ dependencies between species with different successional strategies.

## Introduction

Tropical forests play an important role in controlling the global carbon cycle and, thus, the rate of ongoing climate change (Lewis, 2006; Stocker et al., 2014). They store more than half of the carbon in the world’s forests (Pan et al., 2011), and provide roughly one-third of the global terrestrial primary production (Beer et al., 2010). Reliable quantification of the carbon uptake of tropical forests across time and space thus requires understanding of how carbon fluxes (carbon gain through photosynthesis and carbon loss through respiration) vary among environmental conditions and tree functional types. More specifically, Dynamic Global Vegetation Models (DGVMs) and Earth System Models (ESMs) require accurate representation of the factors controlling variation in the maximum rates of photosynthetic carboxylation (*V*_cmax_) and electron transport (*J*_max_), as well as leaf respiration (Rogers, 2014; Walker et al., 2014). For tropical forests in general and African rainforests and tropical montane forests in particular, much remains to be explored regarding these controls.

Most DGVMs and ESMs employ the photosynthesis model by Farquhar et al. (1980), which represents the variation in *V*_cmax_ and *J*_max_ (at a reference temperature) as either fixed values for different plant functional types or as linear functions of area-based total leaf nitrogen content (N_tot_; Kattge et al., 2009; Thornton et al., 2009; Zaehle et al., 2010; Rogers, 2014; Walker et al., 2014). However, a recent global meta-analysis found that interspecific variation in *V*_cmax_ and *J*_max_ was much more closely related to photosynthetic N use efficiency than to N_tot_ (Ali et al., 2015). Moreover, several studies in tropical rainforests have found that area-based leaf nutrient content (i.e. N, phosphorous) is often a poor predictor of the large interspecific variation in photosynthetic capacity (Coste et al., 2005; van de Weg et al., 2012; Houter and Pons, 2014; Dusenge et al., 2015; Bahar et al., 2016; Hasper et al., 2017). Some of these studies have indicated that the fractional investment of leaf N into compounds maximizing photosynthetic capacity (i.e. *V*_cmax_ and *J*_max_) is a considerably stronger determinant of interspecific variation in *V*_cmax_ and *J*_max_ than N_tot_ (Coste et al., 2005; Dusenge et al., 2015; Hasper et al., 2017). Yet, more research is needed to confirm this pattern since these studies were conducted on seedlings in a greenhouse (Coste et al., 2005) or on a rather small number of rainforest tree species (six in Dusenge et al., 2015; five in Hasper et al., 2017). The strength of the relationship between photosynthesis and N_tot_ may depend on leaf phosphorus (P) content (Reich et al., 2009). However, *V*_cmax_ – N relationships were similarly weak at both high and lower altitude in Rwanda, in spite of leaf P content being twice as high at the higher site (Dusenge et al., 2015). Furthermore, leaf P content and photosynthetic N use efficiency (*V*_cmax_ per unit leaf N) were not correlated in a large study of Andean and Amazonian rainforest species (Bahar et al., 2016).

Part of the reason for why interspecific variation in photosynthetic capacity is often poorly related to total leaf nutrient content may be that species with different successional strategies differ in within-leaf N allocation. Fast-growing and short-lived early-successional (ES) tree species usually make greater investments in N-rich molecules involved in photosynthesis and respiration than slow-growing and long-lived late-successional (LS) tree species, regenerating in low light under tree canopies (Raaimakers et al., 1995; Valladares and Niinemets, 2008; Xiao et al., 2018). In contrast, leaves of LS species are often more long-lived and make larger fractional investments in rather N-poor structural compounds and pigmentation. However, these patterns do not always hold true for tropical tree species. A study with seedlings of 14 rainforest species found that while leaf mass per unit leaf area (LMA) increased with species’ shade tolerance, photosynthetic capacity and N_tot_ content did not systematically change (Coste et al. (2005). Another study on 17 rainforest tree species, reported that photosynthetic capacity decreased with increasing species’ shade tolerance while LMA and N_tot_ did not change (Houter and Pons, 2014). These studies thus suggest that interspecific variation in photosynthetic capacity in tropical trees is often controlled by within-leaf N allocation, but more research is needed to explore the link between within-leaf N allocation strategies and other plant traits (e.g., other leaf traits, life history traits).

A recent study on six tropical montane rainforest tree species indicated that there may be a trade-off involved in within-leaf N allocation, such that ES species with high fractional N investments into compounds that maximize photosynthetic capacity (i.e. *V*_cmax_ and *J*_max_) invest less N into compounds involved in light-harvesting (i.e., chlorophyll and photosystems), and vice versa for LS species (Dusenge et al., 2015). Such differences between ES and LS species are in line with the “carbon gain hypothesis” put forward to explain plant shade-tolerance. It states that shade-tolerant LS species have plant traits that maximize carbon gain under low light conditions (e.g., low respiration and LMA, high chlorophyll content and quantum yield of photosynthesis; Valladares and Niinemets, 2008). However, the study by Dusenge et al. (2015) found that LS species, in spite of indications of higher chlorophyll content, actually had significantly lower quantum yield than ES species. Clearly, more research is needed to better understand the roles played by different plant traits in controlling shade-tolerance in tropical forests (Valladares et al., 2016; Poorter et al., 2019).

Africa harbors 27% of all tropical forests (Scatena et al., 2010) and 13% of all tropical montane forests (elevation > 1000 m a.s.l; Spracklen and Righelato, 2014). However with respect to ecological and biogeochemical understanding of carbon dynamics, the available data on African tropical forests is scarce, mainly due to the lack of an extensive long-term observation network (Lewis et al., 2009). This is particularly the case for mountainous ecosystems (Mountain Research Initiative EDW working group; Pepin et al., 2015). Here, we investigated physiological, chemical and structural properties of leaves in mature individuals belonging to 12 tree species—five ES and seven LS species—growing in one of Africa’s largest remaining tropical montane rainforests, Nyungwe forest in Rwanda. The overall aim of this study was to explore the controls of interspecific variation in photosynthetic capacity and other leaf gas exchange traits in tropical montane rainforest tree species. Based on previous research, the following predictions were tested:

1. ES species have higher photosynthetic capacity (higher *V*_cmax_, and *J*_max_) than LS species;
2. Area-based total leaf N content is a poor predictor of photosynthetic capacity;
3. Successional groups differ in their within-leaf N allocation;
4. There is a trade-off in the allocation of leaf N between investments into compounds maximizing photosynthetic capacity versus compounds maximizing light harvesting;
5. Key predictions of the “carbon-gain hypothesis” do not apply to montane rainforest tree species.

A previous study in Nyungwe forest showed that neither intra- nor interspecific variation in photosynthetic capacity was related to leaf P content (Dusenge et al., 2015), which was not investigated here.

## Materials and Methods

### Study Site and Plant Species

Data were collected on mature trees (**Table 1**) in Nyungwe National Park (2°17—2°49’ S, 29°03—29°29’ E; elevation 1600–2950 m, investigated plots at 1950–2500 m). Nyungwe National Park (hereafter called “Nyungwe”) is located in the southwestern part of Rwanda, Central Africa, within the Albertine Rift ecoregion (Plumptre et al., 2007). Nyungwe covers 1013 km^2^ and forms, together with the contiguous Kibira national park in Burundi, the largest block of tropical mi-elevation montane forest remaining in Africa, with large areas encompassing a mixture of primary and secondary forest due to its disturbance history (Plumptre et al., 2002).

**Table 1:** Description of early-successional (ES) and late-successional (LS) tree species investigated in this study.

Species	Family	Successional group^a^	Diameter at breast height (cm)	%BA in plots	Tree height (m)
*Hagenia abyssinica* (Bruce) J.F.Gmel.	Rosaceae	ES	28 ± 14	0.3	8 ± 5
*Harungana montana* Spralet	Clusiaceae	ES	41 ± 21	2.3	20 ± 2
*Macaranga kilimandscharica* Pax	Euphorbiaceae	ES	22 ± 5	24.8	15 ± 4
*Prunus africana* (Hook. f.) Kalkm.	Rosaceae	ES	35 ± 13	0.6	19 ± 6
*Polyscias fulva* (Hiern.) Harms	Araliaceae	ES	52 ± 13	3.4	20 ± 3
*Carapa grandiflora* Sprague	Meliaceae	LS	40 ± 22	2.6	19 ± 5
*Cleistanthus polystachyus* Hook.f. ex Planch.	Euphorbiaceae	LS	31 ± 15	2.6	18 ± 4
*Faurea Saligna* Harv.	Proteaceae	LS	53 ± 21	6.1	25 ± 7
*Ficalhoa laurifolia* Hiern.	Theaceae	LS	37 ± 12	2.5	22 ± 4
*Ocotea kenyensis* (Chiov.) Robyns & Wilczek	Lauraceae	LS	40 ± 18	3.0	22 ± 5
*Strombosia scheffleri* Engl.	Olacaceae	LS	31 ± 10	1.2	20 ± 6
*Syzygium guineense* (Willd.) DC.	Myrtaceae	LS	50 ± 21	26.6	20 ± 5

Means ± SE are represented for n = 5–7 species per successional group and 7–15 trees per species.

^a^The classification of the species into successional groups was based on information in the following publications in combination with our own observations of abundance in plots dominated by Macaranga kilimandscharica (main ES species) and Syzygium guineense (main LS species) trees: Bloesch et al., 2009; Fischer and Killmann; Bussmann, 2002; Tesfaye et al., 2002; Fashing, 2004; Fashing et al., 2004; Eilu and Obua, 2005; Kindt et al., 2014; Rutten et al., 2015.

At a meteorological station located at Uwinka (2° 28’43”S, 29° 12’00” E, 2465 m altitude; Nsabimana, 2009; Nyirambangutse et al., 2017), the average day and night air temperatures during 2007–2015 were 15.8°C and 13.5°C, respectively, and the difference between the warmest and coldest month was 1.1°C. The mean relative humidity was 84% and annual rainfall was 1855 mm.

Nyungwe harbors more than 260 tree and shrub species, with 24 recorded as endemic to the Albertine rift (Plumptre et al., 2002). The 12 species investigated in this study were selected to represent common ES and LS species, according to data from 15 half ha monitoring plots recently established in the forest (**Table 2** in Nyirambangutse et al., 2017). The most abundant ES and LS species in Nyungwe are *Macaranga kilimandscharica* and *Syzigium guineense*, respectively, each accounting for 18% of the total number of trees with a diameter at breast height ≥ 30 cm according to a forest-wide survey (Plumptre et al., 2002). The other ES species co-occurred with *M.* *kilimandscharica*, except *H. abyssisnica* which was found at edges and gaps, while the five of the six other LS species clearly co-occurred with *S. guineense*. *Ocotea kenyensis* occurred together with both *M.* *kilimandscharica* and *S. guineense* but has been described as a LS species in the literature (Tesfaye et al., 2002). It was present mostly as rather large trees in our plots, indicating that when co-occurring with *M.* *kilimandscharica* it might be a survivor of earlier disturbance events. The 12 studied species together account for 76% of the total basal area of all trees with diameter at breast height ≥5 cm in the 15 monitoring plots (**Table 1**).

**Table 2:** Summary report with results of a two-factor mixed-effects ANOVA and a linear mixed-effects model (see *Statistical Analysis* section).

Parameter	Factor	F-value	p-value
*Mixed-effects ANOVA*			
*V*_cmax25_	Succ	9.8	**0.011**
*J*_max25_	Succ	11.6	**0.009**
*J*_max25_/*V*_cmax25_	Succ	0.2	0.66
A_280_	Succ	9.4	**0.012**
AQY	Succ	10.7	**0.008**
*R*_d25_	Succ	6.9	**0.025**
N_tot_	Succ	0.4	0.54
LMA	Succ	1.4	0.26
Chl	Succ	1.6	0.23
*Linear mixed-effects model*			
*V*_cmax25_	N_tot_	0.67	0.41
	Succ	8.7	**0.015**
	N_tot_*Succ	5.8	**0.018**
	N_LH_	11.7	**0.001**
N_R+B_	Succ	4.2	0.067
	N_LH_*Succ	0.5	0.5
	N_tot_	7.14	**0.009**
R_d25_	Succ	7.14	**0.023**
	N_tot_*Succ	0.15	0.7
N_tot_	LMA	72.2	**<0.001**
	Succ	0.0083	0.93
	LMA*Succ	1.5	0.22
	N_LH_	5.7	**0.019**
AQY	Succ	4.8	0.052
	N_LH_*Succ	2	0.16

Bold numbers represent p < 0.05. Traits analyzed were: maximum rates of Rubisco carboxylation capacity (V_cmax25_, µmol m^-2^ s^-1^) and electron transport (J_max25_, µmol m^-2^ s^-1^) at 25°C; photosynthetic rates at a constant intercellular (C_i_) CO_2_ concentration of 280 ppm (A_280_, µmol m^-2^ s^-1^); the ratio of J_max25_ to V_cmax25_ (J_max25_:V_cmax25_ ratio); leaf mass per unit leaf area (LMA, g m^-2^) and area-based total leaf nitrogen content (N_tot_, g m^-2^); leaf dark respiration measured at 25˚C; apparent quantum yield of photosynthesis (AQY); fractional investments of total leaf N content into compounds maximizing photosynthetic capacity (N_R+B_) and compounds maximizing photosynthetic light-harvesting (N_LH_).

### Leaf Gas Exchange Measurements

Field measurements of leaf gas exchange in mature trees were conducted from late February to early August 2015 between 9:00 and 17:00 h, using two portable leaf gas exchange instruments (LI6400; LI-COR Inc., Lincoln, NE, USA) with the standard 2 cm × 3 cm leaf chamber and a light source (6400-02B LED Light Source). Fully expanded newly mature sun leaves without visible damage were selected and measured for responses of net photosynthetic rate (*A*_n_) to eight CO_2_ concentrations (range 60–2000 μmol mol^-1^; so called *A*-*C*_i_ curves) at a photosynthetic photon flux density (PPFD) of 1800 μmol m^-2^ s^-1^. Then, *A*_n_ was measured at five different levels of PPFD (0, 25, 50, 75, and 100 μmol m^-2^ s^-1^; so called light-response curves) at a CO_2_ concentration of 400 μmol mol^-1^ of air entering the leaf chamber. The relative air humidity was kept between 60% and 80% during the measurements to avoid stomatal closure. Measurements of the response of *A*_n_ to *C*_i_ were performed only if the starting value of stomatal conductance (*g*_s_) was above a minimal threshold of 0.03 mol m^-2^ s^-1^. Measurements of dark respiration (*R*_d_) were conducted on a neighboring leaf, which had been covered by tinfoil and acclimated to darkness for a least 30 min prior to the measurement, to avoid post-illumination CO_2_ burst (Atkin et al., 1998). Most measurements were conducted at a leaf temperature of 20°C, but 12 measurements conducted under unusually warm conditions were made at 25°C.

Leaf gas exchange was measured on one leaf per tree in at least eight trees per species. The trees were selected from as many of the 15 forest plots as possible (some species were, however, present in only a few plots) to account for possible differences among plots (e.g., fertility). The total number of measured leaves was 116. Sun leaves were made accessible by cutting 1 to 2 m branches using a saw mounted on a 20 m long telescopic pole. The branches were immediately placed into a water-bucket prior to gas exchange measurements. The short-term effect of cutting was previously evaluated for Nyungwe tree species, showing no significant effect on *V*_cmax_ and a quite small negative effect (−8%; *p* < 0.05) on *J*_max_ (Dusenge et al., 2015). It was therefore unlikely that branch excision caused a sufficient disruption of xylem water continuity to substantially affect gas exchange measurements, as it may occur in some tropical tree species (Santiago and Mulkey, 2003).

After the measurement campaign from late February to April, it was found that a leak had been present in one of the two instruments used. The conductance of the leak was quantified and used to recalculate *A*_n_ and *C*_i_ data on the assumption that the CO_2_ concentration around the leaf chamber was 400 μmol mol^-1^. Species-specific *V*_cmax_ values determined for the adjusted data were very similar to the *V*_cmax_ values determined for data from the instrument without a leak (on average 1% difference). However, we refrain from reporting *J*_max_ data for the measurements affected by the leak since the leak correction was considerably larger at high compared to low CO_2_ concentrations inside the leaf chamber (e.g., about five times as large at 2000 μmol mol^-1^ than at 60 μmol mol^-1^, at an ambient outside CO_2_ concentration of 400 μmol mol^-1^). As result, we present no *J*_max_ data for two out of 12 species.

### Leaf Gas Exchange Data Analyses

The photosynthesis model by Farquhar et al. (1980), with modifications of photosynthetic temperature dependencies by Bernacchi et al. (2001), was used to parameterize *V*_cmax_ and *J*_max_ from *A*-*C*_i_ curve data by the least squares method. The rates of carboxylation-limited (*A*_c_) and electron transport-limited net photosynthesis (*A*_j_) were calculated as:

(Eqn 1) $$A_{c}=\frac{V_{cmax}(C_{i}-\Gamma^{*})}{C_{i}+K_{c}\left(1+\frac{O}{K_{o}}\right)}-R_{l}$$

and

(Eqn 2) $$A_{j}=J\frac{C_{i}-\Gamma^{*}}{4\;C_{i}+8\Gamma }=R_{l}$$

where *C*_i_ is the leaf intercellular CO_2_ concentration, *K*_c_ and *K*_o_ are Michaelis-Menten constants for CO_2_ and O_2_, respectively; Γ* is the CO_2_ compensation point in the absence of mitochondrial respiration; *R*_l_ is the non-photorespiratory CO_2_ release in the light; and *J* is the rate of electron transport. For *K*_c_, *K*_o_, and Γ*, the values at 25°C as well as the temperature sensitivities were taken from Bernacchi et al. (2001). The internal leaf conductance for CO_2_ was not estimated and therefore “apparent” *V*_cmax_ and *J*_max_ values are reported, based on *C*_i_ rather than on the CO_2_ concentration at the chloroplast. The parameterization of *V*_cmax_ and *J*_max_ were done based on partial pressure units (P_a_) of CO_2_ (*C*_i_ and Γ*) and O_2_; not on mole-based units.

Values of *V*_cmax_, *J*, and *R*_l_ were determined simultaneously with the only *a priori* restriction made to the *A*-*C*_i_ fitting that data points with *C*_i_ below 100 µmol mol^-1^ were forced to be *V*_cmax_-limited. Values of *J*_max_ were estimated from *J* as in Medlyn et al. (2002). The uncertainty of the values of the curvature of the light-response (0.9) and quantum yield of electron transport (0.3 mol electrons mol^-1^ photons) used when calculating *J*_max_ from *J* has only a minor effect on the estimated value of *J*_max_ (Medlyn et al., 2002). Values of *J*_max_ were reported only if the *A*_j_ limited part of the *A*-*C*_i_ curve had at least two data points, or from one single data point if *C*_i_ > 1000 µmol mol^-1^ and/or *A*_j_ was at least 10% lower than *A*_c_ at the *C*_i_ value of that data point. These criteria caused the exclusion of only two *J*_max_ values. Light-saturated net photosynthesis at a common *C*_i_ of 280 µmol mol^-1^ (*A*_280_; assuming the intercellular to ambient CO_2_ concentration to be 0.7) was calculated based on the fitted photosynthesis model for each leaf. Values of *V*_cmax_, *J*_max_, and *A*_280_ are reported for a reference leaf temperature of 25°C using temperature response equations from Bernacchi et al. (2001), although most measurements were conducted at 20°C. Reported values of *R*_d_ were standardized to a leaf temperature of 25°C (*R*_d25_) using a Q_10_ value of 2.14, as suggested for tropical species (Atkin and Tjoelker, 2003).

The apparent (i.e. based on incident rather than absorbed radiation) quantum yield of photosynthesis was determined as the slope of the light-response curve in the PPFD range 25–50 μmol m^-2^ s^-1^.

### Leaf Structural and Chemical Traits

After gas exchange measurements, leaves were collected and the dry mass of leaf discs of known area was recorded after drying at 70°C until constant weight in order to calculate leaf mass per unit leaf area (LMA, g m^-2^). Discs were then ground to fine powder in a ball mill, which was weighed and analyzed for N concentration using an elemental analyzer (EA 1108; Fison Instruments, Rodano, Italy).

Leaves were also measured for SPAD values, a proxy of leaf chlorophyll content (Uddling et al., 2007) optically measured using a SPAD meter (SPAD model 502; Minolta corporation, Ltd., Osaka, Japan). Ten evenly distributed readings were made across each leaf, again avoiding major veins. Leaf chlorophyll content was estimated from SPAD measurements using an equation for tropical tree species provided by Coste et al. (2010).

### Within-Leaf N Allocation

The leaf N investments were determined for the following components of the photosynthetic apparatus: Rubisco (N_R_); bioenergetics, including coupling factors, electron carriers except for photosystems, and Calvin-Benson cycle enzymes except for Rubisco (N_B_); and light-harvesting complexes and photosystems (N_LH_).

The N_R_ was estimated using the equation and parameters provided by (Niinemets and Tenhunen, 1997):

(Eqn 3) $$N_{R}=\frac{0.160V_{cmax}}{V_{cr}}$$

where *V*_cmax_ is the maximum rate of carboxylation, 0.160 converts Rubisco to N [g N in Rubisco (g Rubisco)^-1^] and *V*_cr_ the specific activity of Rubisco at 25°C [20.78 μmol CO_2_ (g Rubisco)^-1^ s^-1^].

The N_B_ was estimated as:

(Eqn 4) $$N_{B}=\frac{J_{max}}{156\times 8.06}$$

where it is assumed that N in bioenergetics is proportional to *J*_max_, that 156 is the ratio of electron transport to cytochrome f content in mol mol^-1^ s^-1^ and that 8.06 is the amount of cytochrome f per unit N in bioenergetics in μmol g^-1^ (Niinemets and Tenhunen, 1997). The sum of N_R_ and N_B_ (N_R+B_) was used as a measure of leaf N in compounds determining the maximum photosynthetic rate, i.e. photosynthetic capacity

The N_LH_ was estimated according to Evans and Poorter (2001) as:

(Eqn 5) $$N_{LH}=41\times 0.0155\times Chl$$

where *Chl* is the area-based chlorophyll content (g m^-2^), 41 is the N content per unit chlorophyll in light-harvesting complexes and photosystems in sun exposed leaves in mol mol^-1^, and 0.0155 is the molar mass ratio of N to chlorophyll. We divided N_R+B_ and N_LH_ by N_tot_ to get the fractional investments (g g^-1^) to compounds maximizing photosynthetic capacity and light harvesting, respectively.

For leaves lacking *J*_max_ data (see *Leaf Gas Exchange Measurements* section above), N_B_ was estimated by assuming that these leaves had the same *J*_max_/*V*_cmax_ ratio as other leaves of the same species for which *J*_max_ data were available. In two species lacking J_max_ data altogether, the J_max_/V_cmax_ ratio was assumed to be the mean of all other species (which did not significantly differ among the other species or between ES and LS species). The fraction of the total leaf N was markedly smaller for N_B_ (4%) than for N_R_ (21%), causing small uncertainty in the estimation of N_R+B_ introduced by this N_B_ data gap filling.

### Statistical Analysis

To analyze the effect of successional identity on photosynthetic capacity (*V*_cmax25_, *J*_max25_ and *J*_max25_/*V*_cmax25_ ratio), *R*_d25_, AQY, LMA, chlorophyll content, and N_tot_, we used a two-factor mixed-effects ANOVA, with successional identity as a main factor and species as a random factor nested within successional group. The relationship between *V*_cmax25_ and N_tot_ was analyzed with a linear mixed-effects model following Zuur et al. (2009) with *V*_cmax25_ as response variable, successional identity as a categorial factor, N_tot_ as a covariate, and species as a random factor with trees as replicates. We had five and seven species for early- and late-successional (**Table 1**), respectively, and for each species 7–15 trees were measured. Differences were considered statistically significant if *p* < 0.05. All analyses were performed in R (version 3.5.2), and the following packages were used: *lme4* (for mixed-effects modelling), *dplyr* (for data manipulation), and *ggplot2* and *cowplot* (for graphing).

## Results

**Figure 1:**
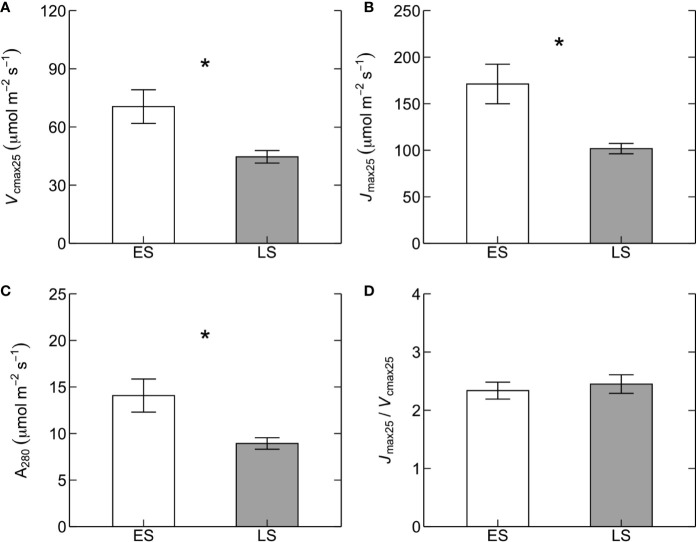
Photosynthetic traits at 25˚C. Maximum rates of **(A)** Rubisco carboxylation capacity (*V*_cmax25_, µmol m^-2^ s^-1^) and **(B)** electron transport (*J*_max25_, µmol m^-2^ s^-1^), **(C)** photosynthetic rates at a constant intercellular CO_2_ concentration (C_i_) of 280 ppm (*A*_280_, µmol m^-2^ s^-1^), and **(D)** the ratio of *J*_max25_ to *V*_cmax25_ (*J*_max25_/*V*_cmax25_) in early-successional (ES, white) versus late-successional (LS, gray) tree species in Nyungwe forest. The asterisks (in **A–C**) indicate statistical significance (*p* < 0.05). Error bars represent SE with n = 5–7 species per successional group and 7–15 trees per species.

**Figure 2:**
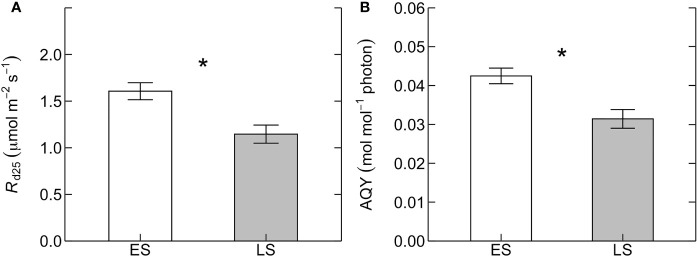
**(A)** Leaf dark respiration measured at 25˚C (*R*_d25_, µmol m^-2^ s^-1^) and **(B)** apparent quantum yield of photosynthesis (AQY, mol mol^-1^ photon) for early-successional (ES, white) and late-successional (LS, gray) tree species in Nyungwe forest. The asterisks (in **A, B**) indicate statistical significance (*p* < 0.05). Error bars represent SE with n = 5–7 species per successional group and 7–15 trees per species.

**Figure 3:**
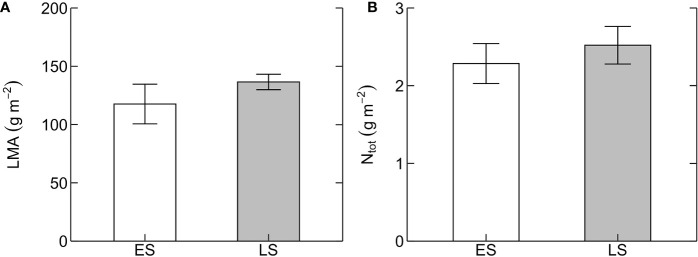
Leaf structural and chemical traits. **(A)** Leaf mass per unit leaf area (LMA, g m^-2^) and **(B)** area-based total leaf nitrogen content (N_tot_, g m^-2^) in early-successional (ES, white) and late-successional (LS, gray) tree species in Nyungwe forest. Error bars represent SE with n = 5–7 species per successional group and 7–15 trees per species.

Basal rates of photosynthetic capacity (i.e. *V*_cmax25_ and *J*_max25_) differed between ES and LS species. *V*_cmax25_ was 58% higher in ES (71 ± 9 µmol m^-2^ s^-1^) than in LS (45 ± 3 µmol m^-2^ s^-1^) species (**Figure 1A**; **Table 2**). Similarly, *J*_max25_ was 68% higher in ES (171 ± 21 µmol m^-2^ s^-1^) than in LS (102 ± 6 µmol m^-2^ s^-1^) species (**Figure 1B**). Values of A_280_ were 58% higher in ES (14 ± 2 µmol m^-2^ s^-1^) than LS (9 ± 1 µmol m^-2^ s^-1^) species (**Figure 1C**). The *J*_max25_/*V*_cmax25_ ratio (2.4 across all species) was not statistically significant between ES and LS species, despite the relatively larger difference in *J*_max25_ compared to *V*_cmax25_ between ES and LS species (**Figure 1D**). Leaf dark respiration at 25˚C (R_d25_) was 33% higher in ES (1.6 ± 0.1 µmol m^-2^ s^-1^) compared to LS species (1.2 ± 0.1 µmol m^-2^ s^-1^) (**Figure 2A**, **Table 2**). Similarly, apparent photosynthetic quantum yield (AQY) was 35% higher in ES (0.042 ± 0.003 mol mol^-1^ photon) than in LS (0.031 ± 0.003 mol mol^-1^ photon) species (**Figure 2B**). Additionally, neither total leaf nitrogen (N_tot_, 2.4 g m^-2^ across all species), nor LMA (128 g m^-2^), nor chlorophyll content (0.84 g m^-2^) differed between ES and LS groups (**Figure 3**; **Table 2**; **Supplementary Table 2**).

**Figure 4:**
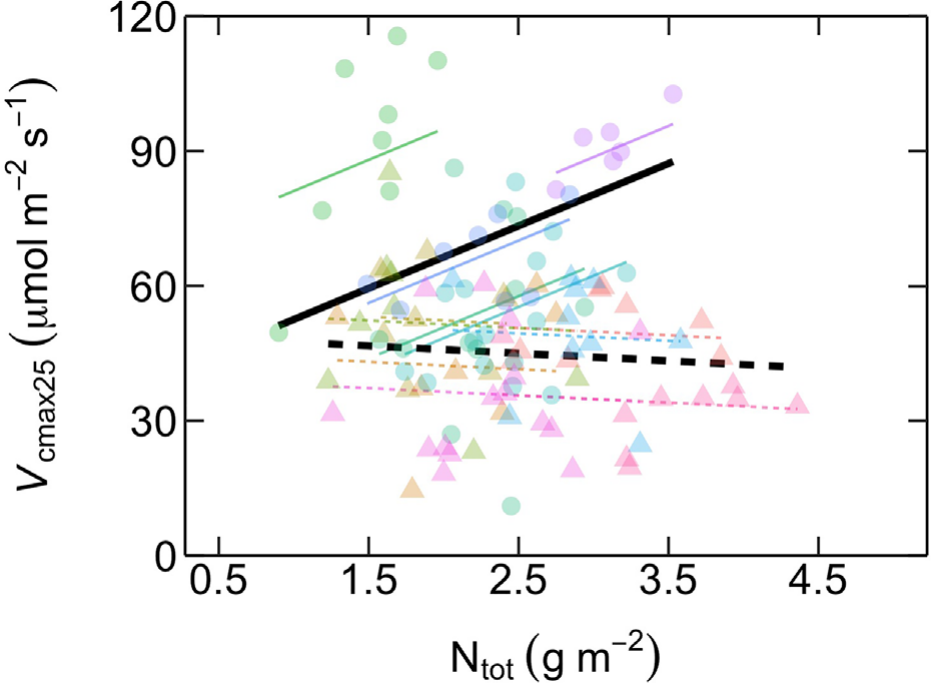
Relationship between maximum rates of Rubisco carboxylation capacity at 25˚C (*V*_cmax25_, µmol m^-2^ s^-1^) as a function of area-based total leaf nitrogen content (N_tot_, g m^-2^) in early-successional (ES) and late-successional (LS) tree species in Nyungwe forest. Different symbol colors represent each of the 12 studied species, and symbol shapes represent successional groups (ES = circle; LS = triangle). Black solid (ES: *V*_cmax25_ = 13.9 N_tot_ + 38.6) and dashed (LS: *V*_cmax25_ = -1.6 N_tot_ + 49.1) lines are overall regression lines for ES and LS groups, respectively. Colored lines represent regression lines for each species belonging to ES (solid) and LS (dashed) groups, respectively, but with common successional group-specific slopes. n = 5–7 species per successional group and 7–15 trees per species.

**Figure 5:**
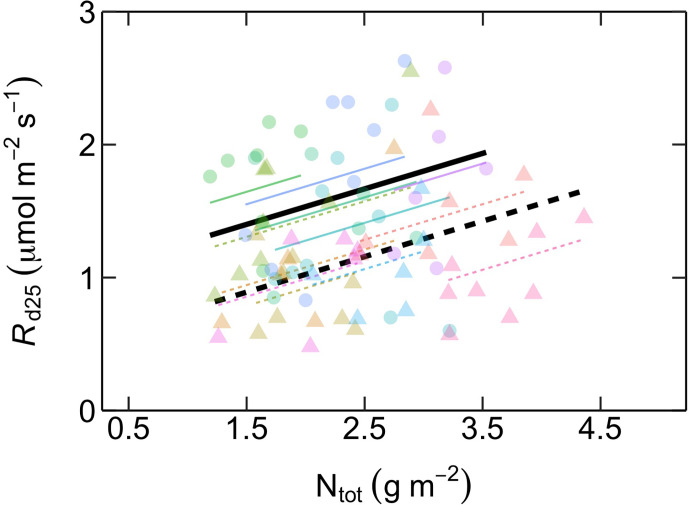
Relationship between dark respiration at 25˚C (*R*_d25_, µmol m^-2^ s^-1^) as a function of area-based total leaf nitrogen content (N_tot_, g m^-2^) in early-successional (ES) and late-successional (LS) tree species in Nyungwe forest. Different symbol colors represent each of the 12 studied species, and symbol shapes represent successional groups (ES = circle; LS = triangle). Black solid (ES: *R*_d25_ = 0.27 N_tot_ + 0.99) and dashed (LS: *R*_d25_ = 0.27 N_tot_ + 0.5) lines are overall regression lines for ES (solid) and LS (dashed) groups, respectively, but with common slopes since these did not significantly differ. Colored lines represent regression lines for each species belonging to ES (solid) and LS (dashed) groups. n = 5–7 species per successional group and 7–15 trees per species.

The relationship between *V*_cmax25_ and N_tot_ differed between ES and LS species (**Figure 4**; **Table 2**). In ES species, *V*_cmax25_ increased with N_tot_, while in LS species there was no such dependency at all. At low N_tot_ (~ 1 g m^-2^), *V*_cmax25_ was similar in both groups. At higher N_tot_, however, ES species had considerably higher *V*_cmax25_ than LS species and this difference increased progressively with the magnitude of N_tot_. However, *R*_d25_ was positively related with N_tot_ for both ES and LS species, with similar slopes but different intercepts (**Figure 5**; **Table 2**).

**Figure 6:**
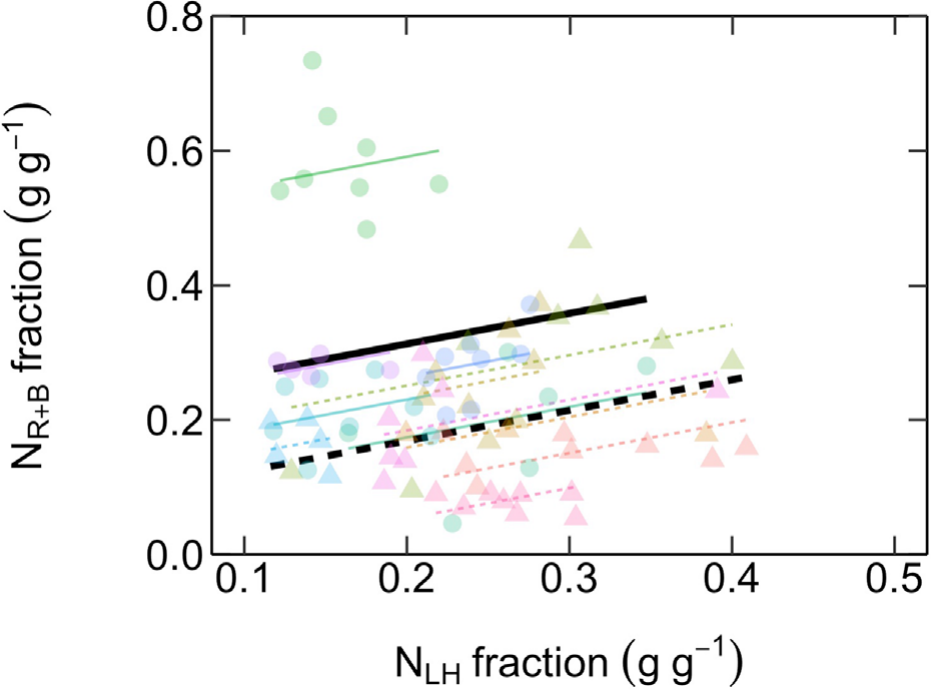
Relationship between fractional investments of total leaf N content into compounds maximizing photosynthetic capacity (N_R+B_, g g^-1^) and compounds maximizing photosynthetic light-harvesting (N_LH_, g g^-1^). Different symbol colors represent each of the 12 studied species, and symbol shapes represent successional groups (ES = circle; LS = triangle). Black solid and dashed lines are overall regression lines for ES (N_R+B_ = 0.45 N_LH_ + 0.22) and LS (N_R+B_ = 0.45 N_LH_ + 0.08) groups, respectively, but with common slopes since these did not significantly differ. Colored lines represent regression lines for each species belonging to ES (solid) and LS (dashed) groups. n = 5–7 species per successional group and 7–15 trees per species.

There was a positive relationship between the fraction of total leaf N investments into compounds maximizing photosynthetic capacity (N_R+B_) versus compounds involved in light-harvesting (N_LH_), with both ES and LS species displaying similar slopes (**Figure 6**, **Table 2**).

For both ES and LS species, N_tot_ was positively related to LMA (**Supplementary Figure 1**; **Table 2**). There was also a negative relationship between N_LH_ and AQY for both groups, with similar slopes and a nearly significantly lower intercept (*p* = 0.052) for LS species (**Supplementary Figure 2**; **Table 2**).

## Discussion

With the overall aim to explore the controls of interspecific variation in photosynthetic capacity in tropical montane rainforest trees, we investigated physiological, morphological and chemical leaf traits in mature trees of 12 Central African montane rainforest tree species with contrasting light requirements. These species together represent about 60% of large trees (stem diameter at breast height ≥ 30 cm) in Nyungwe forest—Africa’s largest remaining mid-elevation montane rainforest block (Plumptre et al., 2002; Chao et al., 2011). The results highlight a marked difference in photosynthetic N dependency between different successional groups, with photosynthetic capacity strongly related to total area-based leaf N content (i.e. N_tot_) in ES species but not in LS species.

Photosynthetic capacity was markedly higher in ES compared to LS species (**Figure 1**). This difference was caused by strongly contrasting relative investments of leaf N to compounds maximizing photosynthetic capacity, since N_tot_ was similar in the two successional groups (**Figure 3**). These results confirm our first and third predictions. The second prediction, of poor dependency of photosynthetic capacity on N_tot_, was true for LS species but not for ES species (**Figure 4**). The lack of significant relationship in LS species is in line with other recent studies on tropical rainforest tree species (e.g. Coste et al., 2005; van de Weg et al., 2012; Houter and Pons, 2014; upland species: Bahar et al., 2016; Hasper et al., 2017; Gvozdevaite et al., 2018). However, it contrasts with a study in lowland forests of subtropical China showing a positive relationship between N_tot_ and photosynthetic capacity in LS species but not in ES species (Zhang et al., 2018). Our results on LS species also differ from those of global meta-analyses demonstrating a significant positive relationship between N_tot_ and photosynthetic capacity in tropical trees (Kattge et al., 2009; Reich et al., 2009; Walker et al., 2014).

There are several possible reasons for why meta-analyses report positive relationships between N_tot_ and photosynthetic capacity while specific field studies do not. First, as we show here, photosynthetic N dependency seems strongly linked to species successional strategy (**Figure 4**). A field study focusing primarily on LS species may therefore not detect any significant relationship between N_tot_ and photosynthetic capacity while a meta-analysis including both ES and LS species would do so. Second, the meta-analyses included several studies that examined the canopy vertical variation in N_tot_ and photosynthetic capacity (Porté and Loustau, 1998; Carswell et al., 2000; Kumagai et al., 2001; Meir et al., 2002; Sholtis et al., 2004; Calfapietra et al., 2005; Domingues et al., 2005; Tissue et al., 2005). Since both N_tot_ and photosynthetic capacity are typically higher in sun leaves than in shade leaves this contributes to the overall relationship between the two variables in these studies (e.g. Carswell et al., 2000). Third, meta-analyses included data from tropical areas with large variation in soil fertility (both N and P) while such variation is considerably lower in most specific field studies. This is well illustrated in a recent field study on 210 tree species from lowland Amazonian (lower soil fertility) and upland Andes (higher soil fertility) tropical rainforests, in which a significant relationship between photosynthetic capacity and leaf N_tot_ was found when data from both sites were pooled together (not necessarily a causal relationship since leaf N and P co-varied; Bahar et al., 2016). However, when trees from upland sites with high and fairly homogenous soil fertility were analyzed alone, no relationship between photosynthetic capacity and N_tot_ was found.

Our findings are in line with a recent global meta-analysis covering all types of plants and ecosystems which showed that within-leaf N allocation was a crucial determinant of variation in photosynthetic capacity (Ali et al., 2015). It further showed that about half of the variation in photosynthetic capacity could be attributed to environmental factors influencing photosynthetic N use efficiency (i.e. *V*_cmax_ or *J*_max_ divided by N content). Our study suggests that successional group is another factor, not included in the meta-analysis of Ali et al. (2015), which may explain a significant part of variation in photosynthetic capacity. Our results suggest ES and LS species allocate equal fractions of leaf N into compounds maximizing photosynthetic capacity at low N_tot_, but that at higher N_tot_ ES species gradually increase their absolute N investments to photosynthetic capacity while LS species do not (**Figure 4**). These results are in agreement with the general understanding of how shade-intolerant ES species and shade-tolerant LS species differ with respect to leaf physiological traits related to carbon assimilation, i.e. that ES species prioritize high photosynthesis and rapid growth (Raaimakers et al., 1995; Hikosaka, 2004; Valladares and Niinemets, 2008; Reich, 2014). They provide novel insight by showing that the typical assumption of N_tot_ as a key determinant of photosynthetic capacity seems to hold for ES species but not for LS species, at least in tropical montane forests.

In contrast to the different relationships between *V*_cmax25_ and N_tot_ in ES and LS species, *R*_d25_ was positively related to N_tot_ in both groups (**Figure 5**). This may reflect that, as N_tot_ increases, LS species invest the additional N at high N_tot_ into maintenance and secondary metabolism (i.e. defense) rather than into increased photosynthetic capacity (which did not increase; **Figure 4**).

The fourth prediction tested—that there is a trade-off in the allocation of leaf N between investments into compounds maximizing photosynthetic capacity versus compounds maximizing light harvesting—was not supported by our results (**Figure 6**). This hypothesis, proposed by Dusenge et al. (2015) and corroborated by Hasper et al. (2017), was based on their observations of a negative relationship between photosynthetic capacity (i.e. V_cmax25_ and J_max25_) and SPAD values (a proxy for area-based leaf chlorophyll content). In the current study, we further tested the hypothesis by explicitly investigating the possibility of a trade-off between fractional leaf N investments into Rubisco and bioenergetics (N_R+B_) and light harvesting compounds (N_LH_). Strikingly, we found the opposite trend, suggesting that the hypothesis of Dusenge et al. (2015) may not be a general trade-off explaining species successional strategy. It is likely that there are other within-leaf N allocation trade-offs involved which were not investigated here. A recent meta-analysis (Onoda et al., 2017) revealed that the trade-off between photosynthetic N and structural N in cell walls, the two major leaf N pools, underlies the “leaf economics spectrum” (Wright et al., 2004; Hikosaka, 2004). However, this type of structure-function trade-off in N allocation is unlikely to explain the differences in the *V*_cmax25_-N_tot_ relationships between ES and LS species found in our study (**Figure 4**), since they did not differ in LMA or N_tot_ (**Figure 3**) and shared a common positive LMA-N_tot_ relationship (SI **Figure 1**).

The fifth prediction tested—that key predictions of the “carbon-gain hypothesis” do not apply to montane rainforest tree species—was corroborated by our study. While photosynthetic capacity and *R*_d25_ (as also seen in Baltzer and Thomas, 2007) differed in a way predicted by the carbon gain hypothesis (both lower in LS species), AQY, N_tot_, chlorophyll content, and LMA did not (**Table 2**). In complete contradiction with that hypothesis, AQY was even lower in LS compared to ES species, as also observed in a previous study on tropical montane trees species (Dusenge et al., 2015). Furthermore, our findings showed a negative relationship between AQY and N_LH_ for both groups, implying that increased allocation of leaf N to light harvesting compounds does not necessarily improve light use efficiency, but rather the opposite. The lack of difference in LMA between LS and ES species was not surprising as it agrees with several studies on both mature and young tropical rainforest trees (e.g. Coste et al., 2005; Houter and Pons, 2014; Dusenge et al., 2015; Mujawamariya et al., 2018; Ntawuhiganayo et al., 2020). Some caution should be taken when interpreting observations on sun-exposed leaves of mature trees with respect to species shade tolerance in the understorey. However, since species ranking of leaf traits potentially linked to shade tolerance appears to be similar in sun and shade leaves of juvenile as well as mature tress (Rozendaal et al., 2006; Coste et al., 2009; Dusenge et al., 2015) our results likely have relevance for trees growing in the shade as well.

### Implications

Most DGVMs and ESMs represent the variation in *V*_cmax_ and *J*_max_ (at a reference temperature) as either fixed values for different plant functional types or as linear functions of area-based leaf N, i.e. N_tot_ (Kattge et al., 2009; Thornton et al., 2009; Zaehle et al., 2010; Rogers, 2014). Our finding of contrasting photosynthetic dependencies on N_tot_ in ES versus LS species suggests that both these approaches are problematic. Constant values for different plant functional types fail to account for factors that control variation in photosynthetic variation within each group, e.g. the variation in N_tot_ of ES species in the present study (**Figure 4**). The N_tot_ function concept, on the other hand, fails to recognize the lack of photosynthetic N dependency found for LS species. Our findings suggest that future model approaches would benefit from introducing a plant trait like within-leaf N allocation or photosynthetic N use efficiency. Such traits may be linked to environmental conditions, as reported earlier (Ali et al., 2015), and also to successional strategy, as found here. Our findings also provide important knowledge to improve the accuracy of smaller-scale process-based models developed to estimate gross and net primary production in tropical montane rainforests. Recent work has supplied these models with a better understanding of the climatic variables and functional traits driving forest productivity, but they still currently suffer from large and unaccounted between-species variation in photosynthetic capacity—leaf N relationships (van de Weg et al., 2014; Fyllas et al., 2017). Additionally, our results suggest that a better understanding of the controls of within-leaf nutrient allocation would contribute to a deeper understanding of plant strategies related to successional status and their position in the “fast-slow” plant economic spectrum (Reich, 2014).

## Data Availability Statement

The datasets generated for this study are available on request to the corresponding author.

## Author Contributions

CZ, MED, GW, and JU designed the study, CZ, MED, EZ, and BN collected the data, and CZ, MD, and JU analyzed the data. CZ, MED, and JU drafted the article using feedback from all co-authors. All authors contributed to the article and approved the submitted version.

## Funding

This study was supported by the Strategic Research Area “Biodiversity and Ecosystem Services in a Changing Climate” (BECC; https://www.becc.lu.se/) and the University of Rwanda—Sweden program for Research, Higher Education and Institutional Advancement, financed by Swedish International Development Cooperation Agency (Sida). Thanks also to IDEA WILD (http://www.ideawild.org) for a field laptop. The first author was also funded by Pôle A2F, Université de Lorraine, France, and the second author was also funded by the European Union’s Horizon 2020 research and innovation programme under the Marie Sklodowska-Curie grant agreement No 844319.

## Conflict of Interest

The authors declare that the research was conducted in the absence of any commercial or financial relationships that could be construed as a potential conflict of interest.
