# Supplementary material for: Contrasting Dependencies of Photosynthetic Capacity on Leaf Nitrogen in Early- and Late-Successional Tropical Montane Tree Species
Source: Front Plant Sci. 2020 Sep 17;11:500479. doi: 10.3389/fpls.2020.500479 (PMC7527595; doi:10.3389/fpls.2020.500479)
Supplement: Supplementary file 1 [file DataSheet_1.pdf]

Contrasting dependencies of photosynthetic capacity on leaf nitrogen in early- and late-successional tropical montane tree species

Camille Ziegler[1,2,3], Mirindi Eric Dusenge[1,4,5], Brigitte Nyirambangutse[1,4], Etienne Zibera[4], Göran Wallin[1], Johan Uddling[1*]

[1] University of Gothenburg, Department of Biological and Environmental Sciences, Gothenburg, Sweden

[2]UMR EcoFoG, CNRS, CIRAD, INRAE, AgroParisTech, Université des Antilles, Université de Guyane, Kourou, France

[3]Université de Lorraine, AgroParisTech, INRAE, UMR Silva, Nancy, France

[4] Department of Biology, University of Rwanda, Huye, Rwanda

[5] Department of Biology, The University of Western Ontario, London, ON, Canada

[*]Correspondence:

Email: johan.uddling@bioenv.gu.se

Phone: +46 (0)31 7863757

Supplementary Table 1. Physiological leaf traits (mean ± SE). Significant group differences ($p < 0.05$) are indicated by different upper-case letters.

**Species**	V_{cmax25} (µmol m^{-2} s^{-1})	J_{max25} (µmol m^{-2} s^{-1})	J_{max}/V_{cmax}	A_{280} (µmol m^{-2} s^{-1})	R_{d25} (µmol m^{-2} s^{-1})	**AQY** (mmol CO_2 mol^{-1} photons)
Hagenia abyssinica	91,8 ± 6.8	224.0 ± 21.5	2.34 ± 0.19	18.3 ± 1.5	1.76 ± 0.15	0.049 ± 0.006
Harungana montana	52,6 ± 6.6	172.6 ± 23.5	2.72 ± 0.06	10.5 ± 1.5	1.50 ± 0.17	0.042 ± 0.002
Macaranga kilimandscharica	52,6 ± 3.6	134.0 ± 15.3	2.53 ± 0.11	10.6 ± 0.8	1.30 ± 0.19	0.036 ± 0.002
Polyscias fulva	90.4 ± 2.7	211.1 [a]	2.24 [a]	18.3 ± 0.7	1.69 ± 0.20	0.043 ± 0.002
Prunus africana	65.5 ± 3.4	114.1 ± 17.3	1.86 ± 0.08	12.6 ± 0.6	1.79 ± 0.23	0.043 ± 0.003
**Early-successional species**	**70.6 ± 8.7 A**	**171.2 ± 21.2 A**	**2.34 ± 0.15**	**14.1 ± 1.8 A**	**1.61 ± 0.09 A**	**0.042 ± 0.003 A**
Carapa grandiflora	50.4 ± 2.5	100.6 ± 20.2	1.93 ± 0.09	9.7 ± 0.5	1.50 ± 0.15	0.034 ± 0.003
Cleistanthus polystachyus	38.9 ± 5.8	106.4 ± 35.7	2.29 ± 0.07	8.5 ± 1.3	1.08 ± 0.20	0.027 ± 0.003

Faurea Salina	52.5 ± 6.7	-	-	10.1 ± 1.5	1.52 ± 0.19	0.038 ± 0.004
Ficalhoa laurifolia	53.1 ± 3.7	-	-	10.7 ± 0.8	0.88 ± 0.10	0.039 ± 0.003
Ocotea kenyensis	49.2 ± 4.1	107.0 ± 14.2	2.49 ± 0.20	10.0 ± 0.8	1.08 ± 0.15	0.032 ± 0.003
Strombosia scheffleri	33.0 ± 2.9	113.7 ± 25.9	2.68 ± 0.18	6.5 ± 0.7	0.98 ± 0.11	0.021 ± 0.002
Syzigium guineense	35.3 ± 3.7	81.3 ± 9.4	2.85 ± 0.10	7.1 ± 0.7	1.45 ± 0.47	0.030 ± 0.003
**Late-successional species**	**44.6 ± 3.2 B**	**101.8 ± 5.5 B**	**2.45 ± 0.16**	**8.9 ± 0.6 B**	**1.21 ± 0.10 B**	**0.031 ± 0.003 B**

[a] Measurements available for one leaf only.

Supplementary Table 2. Structural and chemical leaf traits (mean ± SE). Traits analyzed were: leaf mass per unit leaf area (LMA, g m^{-2}), area-based total leaf nitrogen content (N_{tot}, g m^{-2}) and chlorophyll content (g m^{-2}).

**Species**	**LMA** (g m^{-2})	$\mathbf{N_{tot}}$ (g m^{-2})	**Chlorophyll content** (g m^{-2})
Hagenia abyssinica	68.7 ± 2.5	1.49 ± 0.12	0.37 ± 0.02
Harungana montana	99.1 ± 3.0	2.23 ± 0.12	0.87 ± 0.04
Macaranga kilimandscharica	136.1 ± 6.7	2.39 ± 0.10	0.60 ± 0.03
Polyscias fulva	169.7± 8.5	3.11 ± 0.11	0.68 ± 0.06
Prunus africana	114.5 ± 3.8	2.20 ± 0.16	0.83 ± 0.05
**Early-successional species - Mean**	**117.0 ± 17.03**	**2.29 ± 0.26**	**0.67 ± 0.09**
Carapa grandiflora	166.9 ± 8.6	3.09 ± 0.18	1.45 ± 0.07
Cleistanthus polystachyus	118.5 ± 10.1	2.11± 0.19	0.99 ± 0.06
Faurea Salina	125.3 ± 6.7	1.79 ± 0.18	0.73 ± 0.03

Ficalhoa laurifolia	129.4 ± 8.3	1.97 ± 0.12	0.77 ± 0.04
Ocotea kenyensis	136.1 ± 3.9	2.88 ± 0.13	0.59 ± 0.02
Strombosia scheffleri	125.9 ± 4.0	3.50 ± 0.19	1.49 ± 0.04
Syzigium guineense	153.7 ± 6.6	2.30 ± 0.13	0.71 ± 0.03
**Late-successional species - Mean**	**136.5 ± 6.6**	**2.52 ± 0.24**	**0.96 ± 0.14**

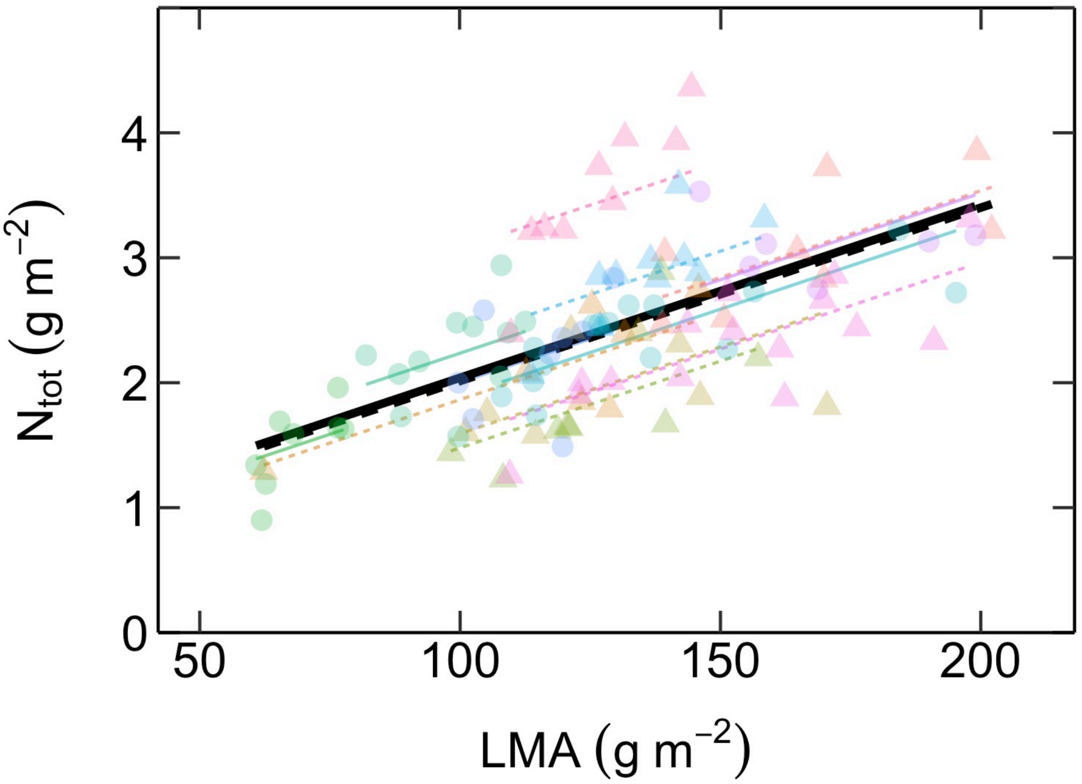

SI Figure 1. Relationship between of area-based total leaf nitrogen content (N_{tot}, g m^{-2}) as a function of leaf mass per unit leaf area (LMA, g m^{-2}) in early-successional (ES) and late-successional (LS) tree species in Nyungwe forest. Different symbol colors represent each of the 12 studied species, and symbol shapes represent successional groups (ES = circle; LS = triangle). Black solid and dashed lines are overall regression lines for ES (N_{tot} = 0.014 LMA + 0.65) and LS (N_{tot} = 0.014 LMA + 0.62) species, respectively, but with common slopes since these did not significantly differ. Colored lines represent regression lines for each species belonging to ES (solid) and LS (dashed) groups. n = 5 – 7 species per successional group and 7 – 15 trees per species.

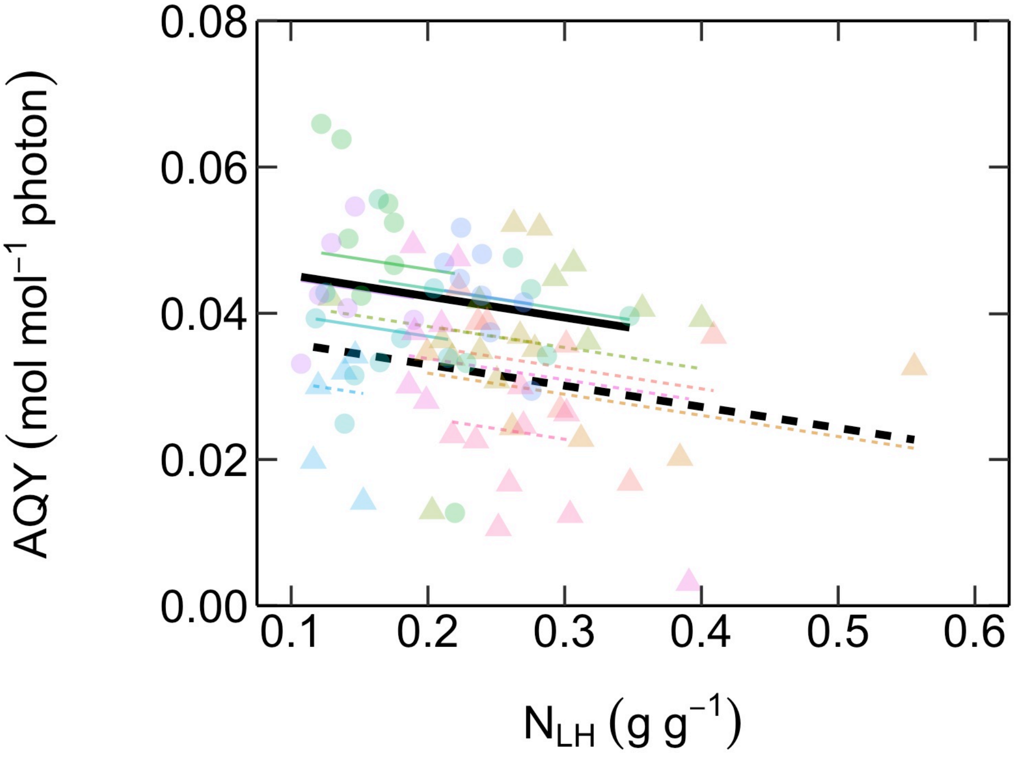

SI Figure 2. Relationship between apparent quantum yield of photosynthesis (AQY, mol mol^{-1} photon) as a function of total leaf N content into compounds maximizing photosynthetic light-harvesting (N_{LH}, g g^{-1}) in early-successional (ES) and late-successional (LS) tree species in Nyungwe forest. Different symbol colors represent each of the 12 studied species, and symbol shapes represent successional groups (ES = circle; LS = triangle). Black solid and dashed lines are overall regression lines for ES ($AQY = -0.029\ N_{LH} + 0.05$) and LS ($AQY = -0.029\ N_{LH} + 0.04$) species, respectively, but with common slopes since these did not significantly differ. Colored lines represent regression lines for each species belonging to ES (solid) and LS (dashed) groups. n = 5 – 7 species per successional group and 7 – 15 trees per species.
